# Chinese Patients’ Willingness to Use Virtual Visits During and After the COVID-19 Pandemic: Repeated Cross-Sectional Study in Zhejiang Province, 2021-2024

**DOI:** 10.2196/87578

**Published:** 2026-10-15

**Authors:** Kaili Chen, Ting Yu, Chengcheng Zhang, Luming Zhao, Liyun Liu

**Affiliations:** 1 School of Humanities and Management Zhejiang Chinese Medical University Hangzhou, Zhejiang China; 2 Integrative Medicine Hospital Zhejiang Chinese Medical University Hangzhou, Zhejiang China

**Keywords:** virtual visit, telemedicine, willingness to use, COVID-19, pandemic

## Abstract

**Background:**

The COVID-19 pandemic has been a critical external driver of telemedicine adoption. As the pandemic entered its later stages, countries worldwide transitioned from stringent containment measures to routine management. This policy shift may in turn have shaped patients’ willingness to use (WTU) telemedicine.

**Objective:**

This study aims to examine the changes in patients’ WTU virtual visits during and after the COVID-19 pandemic in China, with particular attention to the policy transition from stringent containment to routine pandemic management, and to identify factors associated with patients’ WTU across different survey years.

**Methods:**

Repeated cross-sectional surveys were conducted among 340, 786, 1179, and 1373 hospital outpatients in Zhejiang Province, China, from 2021 to 2024. Using year as a proxy for the evolving COVID-19 containment policy context, this study used binary logistic regression of pooled 2021-2024 repeated cross-sectional data to examine whether survey year was significantly associated with patients’ WTU virtual visits. Separate year-specific binary logistic regression was fitted to identify factors associated with patients’ WTU in each survey year.

**Results:**

The proportion of patients’ WTU virtual visits was 70.88% (241/340) in 2021, 71.37% (561/786) in 2022, 62.34% (735/1179) in 2023, and 75.38% (1035/1373) in 2024. In the pooled 2021-2024 repeated cross-sectional analysis, survey year was significantly associated with patients’ WTU: compared with 2021, patients’ likelihood of being willing to use virtual visits was lower in 2023, but higher in 2024. In the year-specific analyses, educational level and primary source of information were consistently associated with patients’ WTU across all 4 years. Visiting hospital tier was additionally associated with WTU in 2022; age and visiting hospital tier were associated with WTU in 2023; and age, monthly income, and self-rated health status were additionally associated with WTU in 2024. Joint Wald tests of year-by-covariate interactions confirmed that the associations of age, visiting hospital tier, monthly income, and self-rated health with WTU varied across survey years.

**Conclusions:**

Patients’ WTU remained high across the 2021-2024 transition from stringent COVID-19 containment to routine pandemic management, declining in the first postpandemic wave of 2023 and then rebounding to its highest level in 2024. The correlates of WTU were only partially stable: a college-level education and internet-based information seeking were consistently associated with higher WTU in both the pooled and year-specific analyses, whereas the associations with age, visiting hospital tier, monthly income, and self-rated health varied across survey years and emerged only in specific periods. These findings suggest that virtual visits gained sustained acceptance among patients in economically and digitally developed regions of China, and that telemedicine platform design should accommodate patients with lower educational levels or limited internet skills while outreach extends across a broader range of age groups to support more equitable adoption.

## Introduction

Since the outbreak of COVID-19 in 2020, the use of telemedicine has increased sharply worldwide [1,2]. According to financial reports from Teladoc Health, one of the leading telemedicine platforms worldwide, the total number of visits in 2020 increased by 156% compared to 2019 [3]. Previous studies also reported that the use of telemedicine in countries such as the United Kingdom and China increased rapidly as a result of the COVID-19 pandemic [4]. Numerous studies have highlighted telemedicine’s advantages during the pandemic, such as lowering cross-infection risks from large-scale in-person visits [5] and enhancing the accessibility of health care services [6]. Consequently, telemedicine emerged as a preferred health care option for an increasing number of patients during the pandemic [7].

As the pathogenicity of the virus weakened and vaccination coverage increased, some countries announced the lifting of emergency restrictions and a return to normal life as early as 2021 [8]. Unlike most countries around the world that lifted their epidemic containment measures between 2021 and 2022, China did so relatively later. In January 2023, China downgraded its COVID-19 containment policies from class A to class B, shifting from strict control measures to routine management [9]. A systematic review on telemedicine use during and after the COVID-19 pandemic reported that as people gradually returned to their prepandemic lifestyles, actual use of telemedicine fluctuated [10]. Within the Expectation Confirmation Model of IS Continuance, initial acceptance (including willingness to use [WTU]) is viewed as the critical first step toward information system success, while continued use ultimately determines its long-term viability [11]. Consistent with this framework, patients’ WTU telemedicine was a predictor of actual use [12]. It is therefore important to examine changes in patients’ WTU telemedicine across COVID-19 containment policy transitions, as such changes may serve as an early indicator of potential shifts in sustained engagement and service sustainability.

In the context of the COVID-19 pandemic, research on patients’ WTU telemedicine has been less extensive than research on actual use. Existing studies on WTU have primarily focused on specific patient populations, such as older adults [13], patients with diabetes [14], and orthopedic patients [15], with few studies based on a broader patient population. Moreover, the majority of existing studies relied on single-year survey data [16-19]; only a limited number of studies have explored changes in patients’ WTU telemedicine before and during the pandemic [13,20,21]. Previous studies have widely adopted the technology acceptance model and the unified theory of acceptance and use of technology to examine how technology acceptance–related factors, such as perceived usefulness, perceived ease of use, social influence, and facilitating conditions, are associated with patients’ WTU telemedicine [22,23]. However, most of these studies relied on single-year cross-sectional survey data and focused on technology acceptance–related factors, which have left the associations between sociodemographic characteristics and WTU telemedicine—particularly whether these associations change over time—largely unexplored. Evidence on how patients’ WTU telemedicine evolved across the COVID-19 containment policy transitions also remains scarce.

China, as a country that maintained COVID-19 containment policies longer than most other countries, offers a unique setting for studying changes in patients’ WTU telemedicine during the COVID-19 pandemic and the post–COVID-19 era. According to the Statistical Report on Internet Development in China released between December 2020 and December 2024, the number of telemedicine users in China showed steady growth until December 2022, but experienced fluctuations between December 2022 and December 2024. Because these fluctuations coincided with the adjustment of COVID-19 containment policies, they underscore that demand for telemedicine did not evolve monotonically around the policy transition and raise the question of whether patients’ WTU telemedicine changed as well. Existing studies conducted in China have explored the general population’s WTU telemedicine in the late stage of the COVID-19 pandemic [24,25]. However, there remains a scarcity of studies on patients’ WTU telemedicine in the post–COVID-19 era. Furthermore, consistent with the international literature, no China-based study has yet examined the association between changes in COVID-19 containment policies and patients’ WTU telemedicine.

In China, virtual visits are a key service type of internet hospitals [26] and are comparable to the telemedicine in previous studies [27]. Assessing patients’ WTU virtual visits therefore provides forward-looking insights into telemedicine adoption. Because China downgraded its COVID-19 containment measures in January 2023—before the September-October 2023 survey wave of this study’s 4 repeated cross-sectional surveys—we classified the 2021-2022 waves as the pandemic period and the 2023-2024 waves as the postpandemic period. Using 4 waves of repeated cross-sectional surveys conducted in Zhejiang Province, an economically and digitally developed region of China, from 2021 to 2024, this study addressed three research questions: (1) What was the annual proportion of patients willing to use virtual visits from 2021 to 2024? (2) Did patients’ WTU virtual visits change across the COVID-19 containment policy transition? (3) What factors were associated with patients’ WTU virtual visits across the pooled 2021-2024 data, and to what extent did the factors associated with WTU remain stable or vary across survey years?

## Methods

### Questionnaire Development and Pilot Testing

The questionnaire items were primarily developed based on previous literature [27-29] and informed by the Health Information National Trends Survey (HINTS) of the US National Cancer Institute [27,30]. HINTS is a systematically developed and validated national survey instrument. Its items have been widely used in studies on digital health. Considering the research objectives and China’s context, we selected and adapted HINTS items related to sociodemographic variables and internet use for the questionnaire. Before the formal survey, the preliminary questionnaire was reviewed by an expert panel consisting of 3 experts (2 clinicians and 1 public health researcher). A pilot survey was conducted on March 1, 2021, involving 25 participants to evaluate the relevance, clarity, and applicability of the questionnaire items. Minor revisions were made based on participant feedback before the formal survey. The completed STROBE (Strengthening the Reporting of Observational Studies in Epidemiology) checklist for this cross-sectional study is provided in Multimedia Appendix 1.

### Participants and Data Collection

In 2021, due to COVID-19 containment measures, we conducted the survey only in Hangzhou, Zhejiang Province. From 2022 to 2024, we used a stratified sampling method to collect survey data across the entire province. Zhejiang, one of the most economically developed provinces in China, has been a pioneer in the digital health sector and a demonstration province in the Internet Plus Healthcare sector. During the COVID-19 pandemic, Zhejiang maintained its position as a leading province in providing virtual visits to its residents by centralizing all local medical resources through a provincial telemedicine platform [27]. Zhejiang has also been at the forefront of applying AI to telemedicine, launching “Anzhen’er”—China’s first government-led digital health companion—which pioneered new models of health care service delivery [31]. “Anzhen’er” has integrated over 30 intelligent health services, connected more than 400 medical institutions across the province, and provided digital health services to over 60 million people [32]. Therefore, a survey of patients in Zhejiang Province, a region with well-developed digital health infrastructure, would not only capture temporal trends in WTU virtual visits from the pandemic to the postpandemic era but also generate region-specific evidence that may inform the scaling of telemedicine services in similar high-income, digitally mature settings.

From 2022 to 2024, repeated cross-sectional surveys were conducted using a multistage stratified sampling design across the 11 prefecture-level cities of Zhejiang Province. Based on per capita gross domestic product (GDP) data from the Zhejiang Statistical Yearbook for the corresponding survey year, the 11 cities were classified into 3 income strata: high-income cities (per capita GDP>150,000 Chinese Yuan [CNY]; 1 CNY=US $0.149 as of September 21, 2026), medium-income cities (150,000 CNY≥ per capita GDP≥100,000 CNY), and low-income cities (per capita GDP<100,000 CNY). Within each stratum, the target sample size was allocated in proportion to the stratum’s share of the total provincial urban population. When selecting survey cities within each income stratum, geographic distribution was also considered to ensure adequate spatial coverage across the province and improve sample representativeness. For example, in the 2024 survey, the target sampling proportions were 39.02% for the high-income stratum (Hangzhou and Ningbo), 21.01% for the medium-income stratum (Shaoxing, Huzhou, and Jiaxing), and 39.97% for the low-income stratum (Taizhou, Jinhua, Lishui, and Wenzhou). The final sample distribution was broadly consistent with the planned sampling proportions. In addition, hospitals of different tiers (primary, secondary, and tertiary) were selected within each income stratum in accordance with China’s 3-tier health care delivery system. Patients were then recruited via convenience sampling in the hospital outpatient departments by trained interviewers. Table 1 presents the basic information of the surveys conducted from 2021 to 2024.

**Table 1:** Basic information on questionnaire surveys from 2021 to 2024.

Survey period	Sampled cities	Sampled hospitals, n	Sample size, n
March-April 2021	Hangzhou	7	366
July-August 2022	High-income city: Hangzhou Medium-income cities: Huzhou, Shaoxing Low-income cities: Jinhua, Lishui, Taizhou	19	883
September-October 2023	High-income cities: Hangzhou, Ningbo Medium-income cities: Shaoxing, Huzhou Low-income cities: Taizhou, Jinhua, Lishui	27	1278
June-July 2024	High-income cities: Hangzhou, Ningbo Medium-income cities: Shaoxing, Huzhou, Jiaxing Low-income cities: Taizhou, Jinhua, Lishui, Wenzhou	30	1424

We conducted questionnaire surveys among hospital outpatients (excluding medical staff) in hospital outpatient waiting areas, using convenience sampling at each participating hospital. All participants completed the questionnaires voluntarily with informed consent. The survey used a paper-based, self-administered format, with research staff available only to distribute and collect forms and to provide clarification when needed. Participants were informed that the survey was completely anonymous, that all responses would be kept confidential, and that the data would be used solely for research purposes. Before data collection, all survey staff received standardized training and were instructed to maintain a neutral attitude throughout the survey, avoiding any leading prompts or suggestions. In addition, all questionnaire items were phrased using neutral wording to minimize the potential influence of social desirability bias. The voluntary and anonymous nature of the survey meant that individuals who declined participation could not be systematically recorded, making it infeasible to calculate a formal response rate.

From 2021 to 2024, a total of 366, 883, 1278, and 1424 questionnaires were collected, respectively. Participants were excluded if they met any of the following criteria: (1) missing sociodemographic information, (2) aged <18 years, (3) not reporting the WTU, or (4) having logically inconsistent responses in other questionnaire items (ie, participants who reported having used virtual visits while also claiming no awareness of virtual visits).

### Measurement

#### Sociodemographic Variables

Based on previous studies and relevant items adapted from the HINTS, the questionnaires used in the 2021-2024 surveys collected sociodemographic variables, including sex, age, self-rated health status, marital status, education level, and monthly income [26-29,32]. To account for the potential confounding effects of hospital tiers (tertiary vs nontertiary hospitals) and city income levels (high-, medium-, and low-income cities), both variables were included as covariates in the regression. Self-rated health status was measured using a 5-point Likert scale, ranging from 1=very poor to 5=very good, and treated as a continuous variable [33]. All other items were defined as categorical variables.

Monthly income was categorized into 5 levels (low, medium-low, medium, medium-high, and high income) based on policy-relevant economic thresholds. Specifically, the low-income category was defined with reference to the minimum wage standard of Zhejiang Province. The remaining categories were determined according to China’s individual income tax thresholds and progressive tax brackets.

#### Primary Source of Information

Based on the internet use items from HINTS, we made contextual adaptations to better reflect China’s context. The original HINTS item asks: “Do you ever go online to access the Internet or World Wide Web, or to send and receive e-mail? (Yes/No) [34].” Given that the internet penetration rate in Zhejiang Province had reached 82.4% by the end of 2020, this binary item was deemed insufficiently discriminating for distinguishing meaningful variation in internet engagement. We therefore modified the item to: “Is the Internet your major source of information?” (Yes/No).

#### WTU Virtual Visits

Patients’ WTU virtual visits was measured via a “yes/no” dichotomous question. Participants were asked the following question: “At present, telemedicine platforms can provide virtual visits, allowing physicians to diagnose and treat certain common and chronic conditions for non–first-visit patients, issue prescriptions, and deliver medications directly to patients’ homes. Would you be willing to use such services?” Participants could choose either “yes” (indicating willingness) or “no” (indicating unwillingness). The questionnaire is available in Multimedia Appendix 2.

### Statistical Analysis Strategy

Stata software (version 18.0; StataCorp LLC) was used to conduct statistical analyses, with significance levels set at *P*<.05. Because the dependent variable was dichotomous (patients’ willingness or unwillingness to use virtual visits), binary logistic regression models were used. All models were estimated with heteroskedasticity-robust SEs to maintain a consistent inferential framework across the pooled and year-specific analyses. First, in the pooled 2021-2024 repeated cross-sectional sample (N=3678), survey year was entered as a set of dummy variables to test whether WTU virtual visits changed over time, using year as a proxy for the evolving COVID-19 containment policy context. Second, year-specific logistic regressions were fitted to each annual cross-section to identify the predictors of WTU within each survey year. Third, to examine whether the association of sociodemographic factors with WTU varied over time, we conducted post hoc joint Wald tests of the interactions between survey year and the covariates whose significance pattern appeared to change across years; these tests were performed in the pooled 2021-2024 repeated cross-sectional data, with survey year specified as a categorical predictor together with all covariates and their interaction terms. To assess the robustness of this study’s findings, we re-estimated the models using hospital-level cluster-robust SEs (Multimedia Appendices 3-5), including city fixed effects (Multimedia Appendix 6), and restricting the sample to the 2022 and 2024 summer waves (Multimedia Appendix 7).

Because the sampling frames varied across the 4 survey years (2021-2024), we conducted a sensitivity analysis restricted to participants from Hangzhou to examine whether the observed annual fluctuations in WTU were sensitive to changes in sample composition. The 2021 wave, fielded during the pandemic when fieldwork was substantially restricted, sampled primarily in Hangzhou; from 2022 onward, as containment measures were relaxed, recruitment was extended to hospitals in high-, medium-, and low-income regions across Zhejiang under a unified sampling strategy to improve representativeness. Annual differences in WTU may thus partly reflect shifts in the sampling frame rather than temporal change alone. To address this issue, we repeated the analysis restricted to Hangzhou—the only city surveyed in all 4 waves—using 1350 valid questionnaires, comparing annual WTU proportions and fitting a binary logistic regression model with survey year entered as indicator variables.

### Ethical Considerations

This study was approved by the medical ethics committee of Zhejiang Chinese Medical University (approval 20210201-2), which was valid for 5 years from February 1, 2021, to February 1, 2026. All participants voluntarily participated in this study after providing oral informed consent. The survey was completed anonymously, and no personally identifiable information was collected. All data were used solely for research purposes and were stored and analyzed in a confidential manner.

## Results

### Participant Characteristics

From 2021 to 2024, a total of 366, 883, 1278, and 1424 questionnaires were collected, respectively. As shown in Figure 1, after the screening process, the final sample sizes in 2021-2024 were 340, 786, 1179, and 1373, corresponding to valid questionnaire rates of 92.90% (340/366), 89.01% (786/883), 92.25% (1179/1278), and 96.42% (1373/1424), respectively. The final pooled sample across 2021-2024 cross-sectional surveys was 3678.

**Figure 1:**
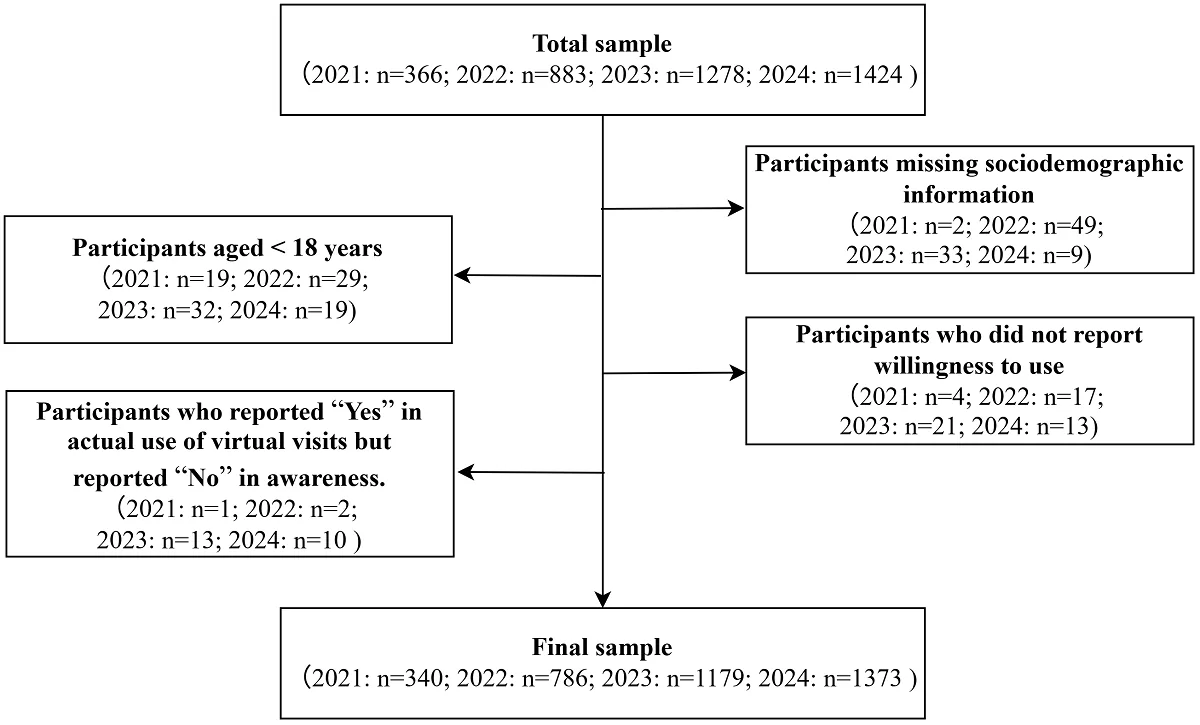
Flow diagram of the final sample selection process.

Table 2 shows the sociodemographic variables of the final sample. Female participants accounted for the majority of patients (2242/3678, 60.96%). The 18- to 29-year age group constituted the largest age subgroup (1330/3678, 36.16%), and college graduates constituted the largest educational subgroup (2056/3678, 55.90%). This demographic skew likely reflected the survey’s urban Zhejiang setting, which tends to attract younger, more educated populations [35]. A total of 82.14% (3021/3678) were in good or fair health. Compared to other variables, monthly income was more evenly distributed across categories. Overall, 74.58% (2743/3678) of the patients reported the internet as their primary source of information.

**Table 2:** Sociodemographic variables of the final sample.

Variable			2021 (n=340), n (%)		2022 (n=786), n (%)		2023 (n=1179), n (%)		2024 (n=1373), n (%)
**Sex**									
	Male	138 (40.59)		314 (39.95)		429 (36.39)		555 (40.42)	
	Female	202 (59.41)		472 (60.05)		750 (63.61)		818 (59.58)	
**Age group (years)**									
	18-29	142 (41.76)		243 (30.92)		489 (41.48)		456 (33.21)	
	30-40	79 (23.24)		228 (29.01)		301 (25.53)		443 (32.27)	
	41-55	77 (22.65)		194 (24.68)		198 (16.79)		300 (21.85)	
	56-65	27 (7.94)		79 (10.05)		107 (9.08)		104 (7.57)	
	>65	15 (4.41)		42 (5.34)		84 (7.12)		70 (5.10)	
**Self-rated health status**									
	Very poor	1 (0.29)		3 (0.38)		2 (0.17)		2 (0.15)	
	Poor	23 (6.76)		31 (3.94)		51 (4.33)		65 (4.73)	
	Fair	132 (38.82)		249 (31.68)		458 (38.85)		563 (41.01)	
	Good	140 (41.18)		356 (45.29)		519 (44.02)		604 (43.99)	
	Very good	44 (12.94)		147 (18.70)		149 (12.64)		139 (10.12)	
**Marital status**									
	Married	207 (60.88)		553 (70.36)		761 (64.55)		955 (69.56)	
	Single	129 (37.94)		216 (27.48)		398 (33.76)		389 (28.33)	
	Divorced	4 (1.18)		14 (1.78)		14 (1.19)		21 (1.53)	
	Widowed	0 (0)		3 (0.38)		6 (0.51)		8 (0.58)	
**Education level**									
	Junior high school or below	61 (17.94)		162 (20.61)		259 (21.97)		286 (20.83)	
	Senior high school	62 (18.24)		191 (24.30)		202 (17.13)		228 (16.61)	
	College graduate	196 (57.65)		409 (52.04)		670 (56.83)		781 (56.88)	
	Postgraduate	21 (6.18)		24 (3.05)		48 (4.07)		78 (5.68)	
**Monthly income**									
	Low income	54 (15.88)		104 (13.23)		197 (16.71)		144 (10.49)	
	Medium low income	133 (39.12)		305 (38.80)		395 (33.50)		338 (24.62)	
	Medium income	63 (18.53)		204 (25.95)		286 (24.26)		442 (32.19)	
	Medium high income	67 (19.71)		144 (18.32)		220 (18.66)		340 (24.76)	
	High income	23 (6.76)		29 (3.69)		81 (6.87)		109 (7.94)	
**Primary source of information**									
	Noninternet	79 (23.24)		172 (21.88)		307 (26.04)		377 (27.46)	
	Internet	261 (76.76)		614 (78.12)		872 (73.96)		996 (72.54)	

### Patients’ WTU Virtual Visits in 2021-2024

Figure 2 showed that patients’ WTU virtual visits fluctuated from 2021 to 2024, with an overall fluctuating upward trajectory across the 4-year period. In 2021, 70.88% (241/340; 95% CI 65.84%-75.45%) of patients were willing to use virtual visits, slightly increasing to 71.37% (561/786; 95% CI 68.11%-74.42%) in 2022. The proportion then declined to 62.34% (735/1179; 95% CI 59.54%-65.06%) in 2023, before rebounding to a 4-year high of 75.38% (1035/1373; 95% CI 73.03%-77.59%) in 2024.

**Figure 2:**
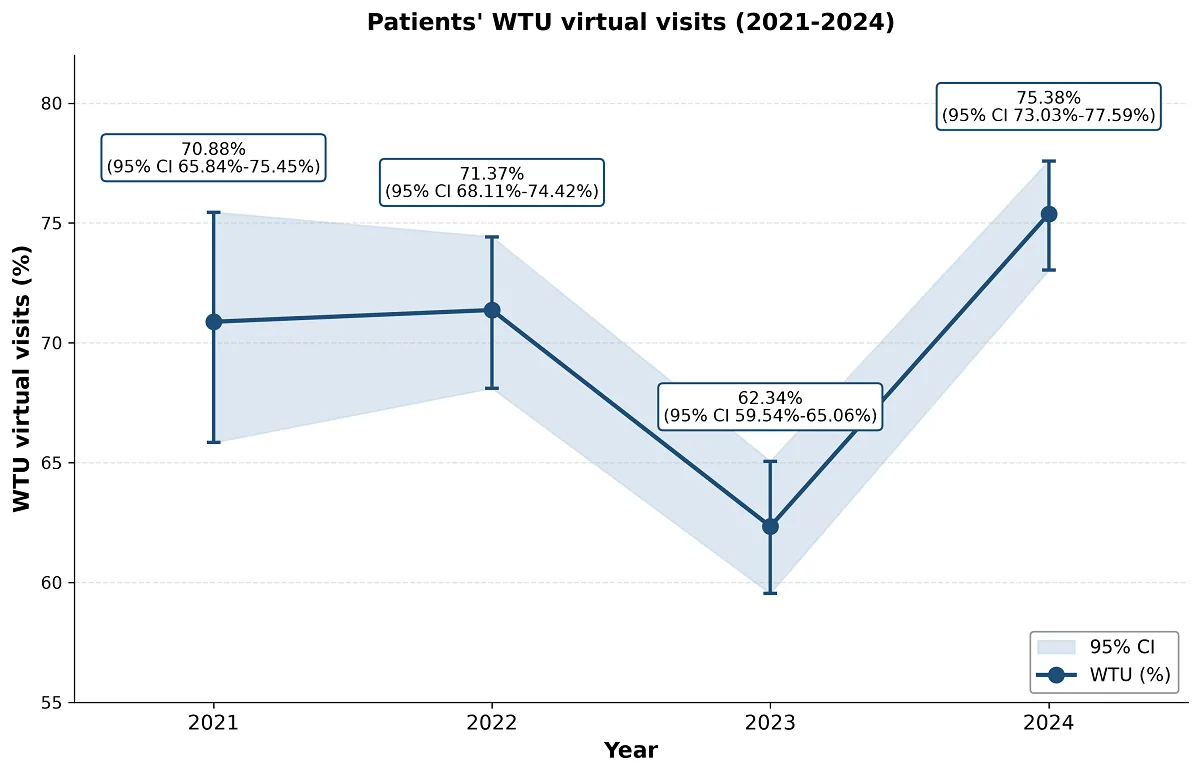
Changes of patients’ WTU virtual visits in 2021-2024. WTU: willingness to use.

### Factors Associated With Patients’ WTU Virtual Visits: Pooled Analysis

The observed fluctuation in patients’ WTU virtual visits may be partly attributable to China’s adjustment of its COVID-19 containment policies in early 2023, when control measures were downgraded from the strictest level to routine management [9]. To examine whether patients’ WTU virtual visits was associated with the COVID-19 containment policy transition, we pooled data from the 4 repeated cross-sectional surveys (N=3678) and fitted a binary logistic regression model with heteroskedasticity-robust SEs, entering survey year as dummy-coded indicator variables (2021 as the reference category) that proxied the policy context in effect when each participant was surveyed. Sensitivity analyses using hospital-clustered robust SEs (46 hospital clusters; Multimedia Appendix 3) and city fixed effects (Multimedia Appendix 6) yielded estimates that were generally consistent with those of the main analysis. To present the regression results consistently across the main analyses, Table 3 reports estimates from the pooled-sample binary logistic regression model fitted with heteroskedasticity-robust SEs.

The results showed that the survey year was significantly associated with patients’ WTU virtual visits. Specifically, compared with 2021, in 2023, patients were less likely to be willing to use virtual visits (odds ratio [OR] 0.71, 95% CI 0.52-0.97; *P*=.03), whereas in 2024, patients were more likely to be willing to use virtual visits (OR 1.42, 95% CI 1.04-1.94; *P*=.03). In contrast, no significant change was observed in 2022 compared with 2021 (*P*>.05). In addition to the survey year, we found that age, education level, monthly income, primary source of information, and visiting hospital tier were also significantly associated with patients’ WTU. The strongest correlates of WTU were higher education and using the internet as the primary information source. Education level showed a dose-response association, with progressively higher odds across senior high school (OR 1.72, 95% CI 1.36-2.18), college (OR 2.74, 95% CI 2.16-3.48), and postgraduate (OR 3.10, 95% CI 1.93-4.98) levels compared with junior high school or below (all *P*<.001). Using the internet as the primary information source was associated with higher WTU (OR 1.70, 95% CI 1.42-2.03; *P*<.001). Two further correlates showed negative associations: attending a nontertiary hospital (OR 0.80, 95% CI 0.66-0.97; *P*=.03), and older age—but only among patients aged ≥56 years (56-65 years: OR 0.40, 95% CI 0.28-0.57; >65 years: OR 0.26, 95% CI 0.17-0.40; both *P*<.001). Monthly income showed a nonlinear association with WTU: only the medium-income group had significantly higher odds than the low-income group (OR 1.36, 95% CI 1.04-1.77; *P*=.03). In contrast, sex, marital status, city income level, and self-rated health status were not significantly associated with WTU in the pooled analysis.

**Table 3:** Binary logistic regression results for factors associated with patients’ WTU virtual visits: pooled 2021-2024 repeated cross-sectional data from Zhejiang Province, China (N=3678)^a^.

Variable		*P* value	OR^b^ (95% CI)
**Sex (reference: male)**			
	Female	.10	1.15 (0.97-1.37)
**Age group (years) (reference: 18-29)**			
	30-40	.66	0.94 (0.71-1.24)
	41-55	.07	0.76 (0.56-1.02)
	56-65	<.001	0.40 (0.28-0.57)
	>65	<.001	0.26 (0.17-0.40)
Self-rated health status		.50	1.04 (0.94-1.15)
**Marital status (reference: married)**			
	Single	.13	1.24 (0.94-1.65)
	Divorced	.55	0.82 (0.43-1.57)
	Widowed	.63	0.72 (0.19-2.78)
**Education level (reference: junior high school or below)**			
	Senior high school	<.001	1.72 (1.36-2.18)
	College graduate	<.001	2.74 (2.16-3.48)
	Postgraduate	<.001	3.10 (1.93-4.98)
**Monthly income level ( reference: low income)**			
	Medium low income	.06	1.27 (0.99-1.63)
	Medium income	.03	1.36 (1.04-1.77)
	Medium high income	.60	1.08 (0.80-1.46)
	High income	.28	1.25 (0.84-1.87)
**Primary source of information (reference: noninternet)**			
	Internet	<.001	1.70 (1.42-2.03)
**Hospital tier (reference: tertiary hospitals)**			
	Nontertiary hospitals	.03	0.80 (0.66-0.97)
**City income level (reference: high-income cities)**			
	Medium-income cities	.43	0.92 (0.73-1.14)
	Low-income cities	.74	0.97 (0.81-1.17)
**Year (reference: 2021)**			
	2022	.31	1.19 (0.85-1.67)
	2023	.03	0.71 (0.52-0.97)
	2024	.03	1.42 (1.04-1.94)
Constant		.21	0.71 (0.41-1.22)

^a^The results are based on heteroskedasticity-robust SEs. McFadden pseudo-*R*^2^=0.1365; Hosmer-Lemeshow *χ*^2^_8_=12.0; *P*=.15; maximum variance inflation factor=3.63. Self-rated health status was measured on a 5-point Likert scale; higher scores indicate better self-rated health status.

^b^OR: odds ratio.

### Year-Specific Factors Associated With Patients’ WTU Virtual Visits

As significant differences across survey years were observed in the pooled analysis, we repeated the binary logistic regression separately for each year to examine whether the correlates of WTU virtual visits were stable or time-varying. Table 4 shows year-specific binary logistic regression results for factors associated with patients’ WTU virtual visits. All year-specific models used the same covariate set and heteroskedasticity-robust SEs as the pooled-sample model; re-estimation with hospital-level cluster-robust SEs yielded generally consistent results (Multimedia Appendices 4 and 5), supporting the robustness of the year-specific estimates.

In 2021, patients with a senior high school education (OR 3.15, 95% CI 1.26-7.84; *P*=.01) or college graduation (OR 3.48, 95% CI 1.33-9.12; *P*=.01) were more likely to be willing to use virtual visits than patients with a junior high school education or below. Patients whose primary source of information was the Internet (OR 2.76, 95% CI 1.43-5.31; *P*=.002) were also more likely to be willing to use virtual visits.

In 2022, patients with a college graduation (OR 1.97, 95% CI 1.16-3.32; *P*=.01) were more likely to be willing to use virtual visits than patients with a junior high school education or below. Patients whose primary source of information was the internet (OR 1.79, 95% CI 1.20-2.67; *P*=.004) were more likely to be willing to use virtual visits. Patients visiting nontertiary hospitals (OR 0.56, 95% CI 0.36-0.86; *P*=.007) were less likely to be willing to use virtual visits than those visiting tertiary hospitals.

In 2023, patients aged 56-65 (OR 0.35, 95% CI 0.19-0.64; *P*=.001) and those older than 65 (OR 0.35, 95% CI 0.17-0.72; *P*=.004) were less likely to be willing to use virtual visits compared with those aged 18-29. Patients with a senior high school education (OR 1.84, 95% CI 1.20-2.83; *P*=.005), a college graduation (OR 2.84, 95% CI 1.86-4.36; *P*<.001), or a postgraduate degree (OR 4.26, 95% CI 1.83-9.89; *P*=.001) were more likely to be willing to use virtual visits compared with patients with a junior high school education or below. Patients whose primary source of information was the internet (OR 1.92, 95% CI 1.39-2.65; *P*<.001) were also more likely to be willing to use virtual visits. Patients visiting nontertiary hospitals (OR 0.65, 95% CI 0.46-0.92; *P*=.01) were less likely to be willing to use virtual visits compared with those visiting tertiary hospitals.

In 2024, patients aged 30-40 years (OR 0.59, 95% CI 0.35-0.99; *P*=.046), 41-55 years (OR 0.51, 95% CI 0.29-0.89; *P*=.02), 56-65 years (OR 0.25, 95% CI 0.13-0.49; *P*<.001), and 65 years and older (OR 0.05, 95% CI 0.02-0.12; *P*<.001) were less likely to be willing to use virtual visits than patients aged 18-29 years. Patients who rated their health as better (coded from 1=very poor to 5=very good) were more likely to be willing to use virtual visits (OR 1.37, 95% CI 1.13-1.67; *P*=.002). Patients with a senior high school education (OR 2.09, 95% CI 1.37-3.20; *P*=.001), a college (OR 3.41, 95% CI 2.28-5.10; *P*<.001), or a postgraduate degree (OR 3.21, 95% CI 1.46-7.08; *P*=.004) were more likely to be willing to use virtual visits than patients with a junior high school education or below. Compared with patients with low monthly income, those with medium-low monthly income (OR 1.68, 95% CI 1.04-2.70; *P*=.03) and medium monthly income (OR 1.74, 95% CI 1.08-2.80; *P*=.02) were more likely to be willing to use virtual visits. Patients whose primary source of information was the internet (OR 1.45, 95% CI 1.07-1.97; *P*=.02) were more likely to be willing to use virtual visits.

Both the pooled 2021-2024 analysis and year-specific analyses showed that using the internet as the primary source of information and having a college-level education were consistently associated with higher WTU virtual visits. By contrast, the associations for certain covariates (ie, age, visiting hospital tier, monthly income, and self-rated health status) appeared to vary across survey years. To formally evaluate these patterns, we performed post hoc joint Wald tests of the interactions between survey year and these 4 covariates in the 2021-2024 pooled-sample logistic regression model (N=3678). All 4 interactions were statistically significant (age×year: Wald *χ*^2^_12_=72.7; *P*<.001; visiting hospital tier×year: Wald *χ*^2^_3_=27.8; *P*<.001; income×year: Wald *χ*^2^_12_=76.5; *P*<.001; self-rated health status×year: Wald *χ*^2^_3_=37.2; *P*<.001). After Bonferroni correction (α=0.0125), all 4 interactions remained significant, confirming that these associations differed across survey years. In the year-specific models, older age was associated with lower WTU only in 2023-2024; attending nontertiary hospitals with lower WTU only in 2022-2023; medium-low income, medium income, and better self-rated health status with higher WTU only in 2024; and sex, marital status, and city income level were not associated with WTU in any survey year.

**Table 4:** Year-specific binary logistic regression results for factors associated with patients’ WTU virtual visits, 2021-2024^a^.

Variable		2021 (n=340), OR^b^ (95% CI)	2022 (n=786), OR (95% CI)	2023 (n=1179), OR (95% CI)	2024 (n=1373), OR (95% CI)
**Sex (reference: male)**					
	Female	0.92 (0.51-1.69)	1.02 (0.72-1.44)	1.18 (0.88-1.59)	1.26 (0.92-1.72)
**Age** **group (years)** **(reference: 18-29)**					
	30-40	1.52 (0.57-4.07)	1.68 (0.90-3.12)	0.92 (0.58-1.44)	0.59 (0.35-0.99)^c^
	41-55	2.02 (0.66-6.24)	1.04 (0.54-2.02)	0.67 (0.41-1.11)	0.51 (0.29-0.89)^c^
	56-65	0.54 (0.15-1.87)	0.86 (0.40-1.85)	0.35 (0.19-0.64)^d^	0.25 (0.13-0.49)^e^
	>65	1.51 (0.37-6.08)	0.64 (0.26-1.59)	0.35 (0.17-0.72)^d^	0.05 (0.02-0.12)^e^
Self-rated health status		0.83 (0.59-1.19)	0.97 (0.78-1.20)	0.99 (0.83-1.19)	1.37 (1.13-1.67)^d^
**Marital status (reference: married)**					
	Single	1.61 (0.62-4.21)	1.26 (0.68-2.32)	1.37 (0.87-2.17)	1.08 (0.64-1.85)
	Divorced	N/A^f^	0.46 (0.13-1.64)	0.40 (0.10-1.59)	1.46 (0.56-3.79)
	Widowed	N/A	0.60 (0.06-6.56)	3.42 (0.69-17.05)	0.19 (0.01-5.89)
**Education level (reference: junior high school or below)**					
	Senior high school	3.15 (1.26-7.84)^c^	1.05 (0.64-1.73)	1.84 (1.20-2.83)^d^	2.09 (1.37-3.20)^d^
	College graduate	3.48 (1.33-9.12)^c^	1.97 (1.16-3.32)^c^	2.84 (1.86-4.36)^e^	3.41 (2.28-5.10)^e^
	Postgraduate	4.61 (0.98-21.57)	2.33 (0.71-7.71)	4.26 (1.83-9.89)^d^	3.21 (1.46-7.08)^d^
**Monthly income level (reference: low income)**					
	Medium low income	1.70 (0.78-3.73)	1.20 (0.70-2.06)	0.97 (0.65-1.46)	1.68 (1.04-2.70)^c^
	Medium income	1.45 (0.60-3.52)	1.21 (0.66-2.22)	1.15 (0.74-1.80)	1.74 (1.08-2.80)^c^
	Medium high income	0.80 (0.33-1.94)	1.02 (0.53-1.96)	1.01 (0.62-1.66)	1.27 (0.73-2.22)
	High income	0.86 (0.25-2.87)	1.06 (0.35-3.22)	0.96 (0.51-1.83)	2.00 (0.95-4.19)
**Primary source of information (reference: noninternet)**					
	Internet	2.76 (1.43-5.31)^d^	1.79 (1.20-2.67)^d^	1.92 (1.39-2.65)^e^	1.45 (1.07-1.97)^c^
**Hospital tier (reference: tertiary hospitals)**					
	Nontertiary hospitals	1.50 (0.79-2.85)	0.56 (0.36-0.86)^d^	0.65 (0.46-0.92)^c^	1.20 (0.81-1.78)
**City income level (reference: high-income cities)**					
	Medium-income cities	—^g^	1.19 (0.70-2.02)	0.90 (0.63-1.30)	0.84 (0.57-1.23)
	Low-income cities	—	0.87 (0.60-1.26)	1.15 (0.85-1.57)	0.79 (0.57-1.10)
Constant		0.36 (0.07-1.91)	1.14 (0.37-3.46)	0.56 (0.24-1.30)	0.57 (0.22-1.49)

^a^The results are based on heteroskedasticity-robust SEs. 2021: McFadden pseudo-*R*^2^=0.1173; Hosmer-Lemeshow *χ*^2^_8_=13.3; *P*=.10; maximum variance inflation factor=4.09; 2022: McFadden pseudo-*R*^2^=0.0782; Hosmer-Lemeshow *χ*^2^_8_=10.8; *P*=.21; maximum variance inflation factor=3.02; 2023: McFadden pseudo-*R*^2^=0.1575; Hosmer-Lemeshow *χ*^2^_8_=2.6; *P*=.96; maximum variance inflation factor=3.03; 2024: McFadden pseudo-*R*^2^=0.1990; Hosmer-Lemeshow *χ*^2^_8_=12.1; *P*=.15; maximum variance inflation factor=3.27. Self-rated health status was measured on a 5-point Likert scale; higher scores indicate better self-rated health status.

^b^OR: odds ratio.

^c^*P*<.05.

^d^*P*<.01.

^e^*P*<.001.

^f^N/A: not applicable.

^g^Not available.

### Sensitivity Analysis

Figure 3 illustrates the comparison of patients’ WTU between Hangzhou and Zhejiang Province from 2021 to 2024. In 2021, the WTU of Hangzhou and Zhejiang Province was identical at 70.88% (241/340; 95% CI 65.84%-75.45%), as the provincial survey was conducted exclusively in Hangzhou that year. From 2022 onward, when the survey expanded to additional cities across Zhejiang Province, Hangzhou consistently exhibited a higher WTU than the provincial average. In 2022, WTU in Hangzhou was 72.90% (191/262; 95% CI 67.69%-77.55%) compared with 71.37% (561/786; 95% CI 68.11%-74.42%) for Zhejiang Province. In 2023, both Hangzhou and Zhejiang Province experienced a decline, falling to 64.12% (252/393; 95% CI 58.89%-69.03%) and 62.34% (735/1179; 95% CI 59.54%-65.06%), respectively. In 2024, both rebounded to their 4-year highs, with Hangzhou reaching 79.44% (282/355; 95% CI 74.96%-83.29%) and Zhejiang Province at 75.38% (1035/1373; 95% CI 73.03%-77.59%). Overall, both Hangzhou and Zhejiang Province demonstrated a similar fluctuating trend over the 4-year period, characterized by a moderate increase from 2021 to 2022, a notable decline in 2023, and a strong rebound in 2024. The parallel trajectory suggested that common temporal factors—most notably the adjustment of COVID-19 containment policies in early 2023—may have similarly predicted patients’ WTU virtual visits across the province.

**Figure 3:**
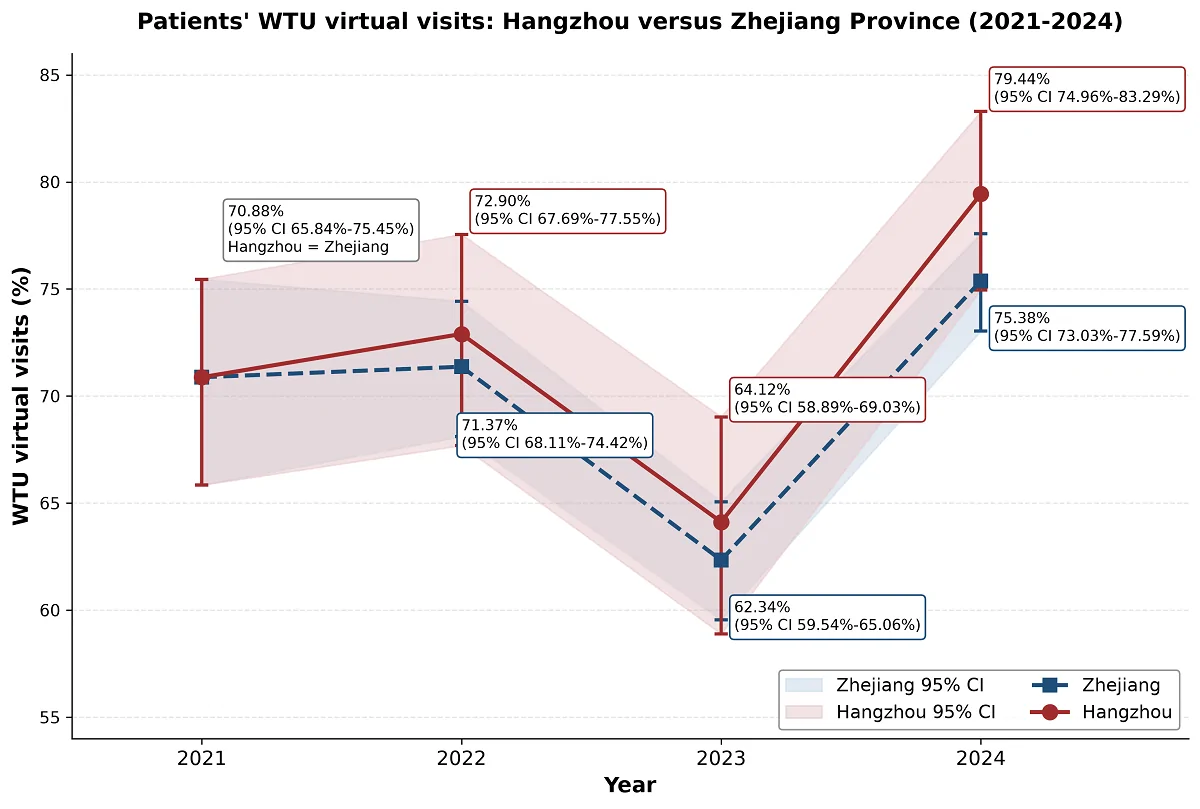
Comparison of patients’ WTU virtual visits between Hangzhou and Zhejiang Province from 2021 to 2024. WTU: willingness to use.

In the logistic regression analysis restricted to the Hangzhou sample (Table 5), the estimate for 2023 was not statistically significant, although the OR remained below 1 (OR 0.77, 95% CI 0.54-1.11; *P*=.16), consistent in direction with the main analysis. In contrast, the OR for 2024 remained significantly greater than 1 (OR 1.64, 95% CI 1.12-2.39; *P*=.01), indicating a higher likelihood of WTU than in 2021, which was also consistent with the main analysis. These findings suggested that the overall direction of the temporal pattern in patients’ WTU observed in the main analysis was broadly consistent in the Hangzhou-only sensitivity analysis. However, the association for 2023 was no longer statistically significant, indicating that the magnitude and statistical significance of the year-specific associations may have been affected by differences in the analytical sample. Nevertheless, the potential influence of changes in the sampling frame cannot be completely ruled out, which was acknowledged as a limitation of this study.

**Table 5:** Binary logistic regression results for factors associated with patients’ WTU virtual visits: pooled 2021-2024 repeated cross-sectional data from Hangzhou, China (n=1350)^a^.

Variable		*P* value	OR^b^ (95% CI)
**Sex (reference: male)**			
	Female	.58	1.08 (0.81-1.44)
**Age group (years) (reference: 18-29)**			
	30-40	.27	1.32 (0.81-2.15)
	41-55	.88	1.04 (0.61-1.77)
	56-65	.06	0.56 (0.31-1.02)
	>65	<.001	0.27 (0.13-0.55)
Self-rated health status		0.66	0.96 (0.81-1.15)
**Marital status (reference: married)**			
	Single	.17	1.40 (0.87-2.27)
	Divorced	.60	0.71 (0.20-2.50)
	Widowed	.32	0.27 (0.02-3.59)
**Education level (reference: junior high school or below)**			
	Senior high school	<.001	2.35 (1.53-3.62)
	College graduate	<.001	3.82 (2.43-5.99)
	Postgraduate	<.001	3.52 (1.77-7.00)
**Monthly income level (reference: low income)**			
	Medium low income	.12	1.43 (0.92-2.22)
	Medium income	.40	1.22 (0.77-1.94)
	Medium high income	.70	0.91 (0.56-1.48)
	High income	.98	1.01 (0.56-1.81)
**Primary source of information (reference: noninternet)**			
	Internet	.001	1.66 (1.22-2.27)
**Hospital tier (reference: tertiary hospitals)**			
	Nontertiary hospitals	.11	0.78 (0.57-1.06)
**Year (reference: 2021)**			
	2022	.37	1.20 (0.80-1.80)
	2023	.16	0.77 (0.54-1.11)
	2024	.01	1.64 (1.12-2.39)
Constant		.22	0.58 (0.25-1.38)

^a^The results are based on heteroskedasticity-robust SEs. McFadden pseudo-*R*^2^=0.1468; Hosmer-Lemeshow *χ*^2^_8_=2.5; *P*=.96; maximum variance inflation factor=3.63. Self-rated health status was measured on a 5-point Likert scale; higher scores indicate better self-rated health status.

^b^OR: odds ratio.

## Discussion

### Principal Findings

Using repeated 2021 to 2024 cross-sectional surveys from an economically and digitally developed region of China, this study examined whether patients’ WTU virtual visits changed across the transition from stringent COVID-19 containment to routine pandemic management and whether its correlates were stable or time-varying. Patients’ WTU remained persistently high across the 4 waves but changed nonmonotonically: after remaining broadly stable between 2021 and 2022, it declined in 2023—the first postpandemic wave—and then rebounded to its highest level in 2024. In the pooled 2021-2024 analysis, survey year was significantly associated with WTU, indicating that WTU differed between the pandemic and postpandemic periods.

Beyond survey year, the correlates of WTU comprised a stable core and a time-varying periphery. A college-level education and using the internet as the primary source of information were consistently associated with a higher WTU, in the pooled-sample model and in every year-specific model, and were among the strongest correlates throughout. By contrast, the associations with age, visiting hospital tier, monthly income, and self-rated health status varied across survey years. Specifically, older age was associated with lower WTU only in the postpandemic waves, with the negative association strengthening and extending to progressively younger age groups by 2024; visiting a nontertiary hospital was associated with lower WTU than attending a tertiary hospital only in 2022-2023; medium-low and medium monthly income and better self-rated health status were associated with higher WTU only in 2024. Joint Wald tests of the year-by-covariate interactions confirmed that these associations differed significantly across survey years, indicating genuine changes in the correlates of WTU over time rather than differences in statistical power between waves. Sex, marital status, and city income level were not associated with WTU in any wave.

Because the sampling frame was expanded beyond Hangzhou after 2021, we also restricted the analysis to Hangzhou—the only city surveyed in all 4 waves—to assess whether the temporal pattern reflected the sampling frame change. The results were largely consistent with the main analysis: the higher WTU in 2024 remained statistically significant, whereas the 2023 decline was attenuated and no longer reached significance. Because the 2021 wave was fielded exclusively in Hangzhou, where digital health infrastructure, concentration of tertiary hospitals, and per capita income are above the provincial average, the reference level of WTU may have been relatively high, which could bias estimates for the subsequent waves downward. The overall temporal pattern therefore appears robust, but the magnitude of the 2023 decline should be interpreted with caution.

### Comparison With Prior Work

#### Patients’ WTU Virtual Visits Before and After China’s COVID-19 Containment Policy Shift

To the best of our knowledge, this study was the first to investigate Chinese patients’ WTU virtual visits during and after the COVID-19 pandemic. The temporal pattern we observed—broadly stable between 2021 and 2022, a decline in 2023, and a rebound to the highest level in 2024—paralleled the telemedicine use trends documented in the China Internet Network Information Center’s Statistical Report on Internet Development in China (2021-2024), suggesting that patients’ WTU changed alongside the adjustment of China’s COVID-19 containment policies rather than evolving independently of it. Notably, the WTU levels in our hospital-based sample (2021: 241/340, 70.88% and 2022: 561/786, 71.37%) were lower than the over 90% acceptance reported by a web-based cross-sectional survey of the general public conducted in China during 2021-2022 [25]. This discrepancy may be attributable to differences in survey methods and sample sources. As the previous study was conducted using an online questionnaire, its participants may have had higher levels of digital literacy and greater experience with internet use, making them more likely to accept telemedicine services.

The high and stable WTU during 2021-2022 was consistent with the pandemic context. Under stringent containment measures, virtual visits were widely regarded as a safer alternative to face-to-face care because they reduced the risk of infection [5,7]. The rise in COVID-19 cases during the pandemic not only led to a shortage of in-person medical visits [36] but also amplified public concerns about infection risks associated with in-person medical visits [37]. These factors may have contributed to the increased demand for virtual visits in China. Consistent with this interpretation, a study conducted in China showed that higher frequency of telemedicine use during the pandemic was associated with increasingly positive public attitudes toward telemedicine [36]. Furthermore, at the beginning of the COVID-19 pandemic, the Chinese government issued a series of policies strongly supporting virtual visits as a response to the epidemic. Policies included establishing a provincial-level internet medical service platform [38], encouraging telemedicine platforms to provide free consultation services [39], and granting virtual visits the authority to prescribe prescription drugs for COVID-19 [40]. In particular, expanded insurance coverage for virtual visits reduced patients’ out-of-pocket expenses [41], which may have lowered the financial barrier to adopt virtual visits.

However, this high and stable pattern did not persist into 2023. Patients’ WTU dropped to 62.34% (735/1179) in 2023, which was consistent with the logistic regression results showing a significant negative association between the year 2023 and patients’ WTU. As China downgraded its COVID-19 containment measures in early 2023, hospital outpatient services gradually resumed [9]. A large number of studies have shown that during the pandemic in 2021-2022, a substantial proportion of nonemergency medical service needs were delayed, such as routine physical examinations, chronic disease treatment, and elective surgeries [42-44]. The subsequent release of patients’ pent-up demand for in-person care after the pandemic may have partly explained the decline in patients’ WTU virtual visits in 2023. This interpretation was consistent with national statistics of the Statistical Bulletin on the Development of Health Care in China (2023): total visits to medical and health institutions nationwide reached 9.55 billion in 2023, an increase of 1.13 billion visits (13.5%) compared with 2022 [45], far exceeding the growth rate observed in other years. Additionally, China experienced a nationwide COVID-19 infection wave following the relaxation of containment policies [46], which may have further contributed to the decline in patients’ WTU in the second half of 2023. This finding was also consistent with a postpandemic survey of health care preferences among 2080 adults in the United States, which found that although 66.5% of respondents preferred to retain at least some telehealth visits in future care, 53% still favored in-person visits when both options were available [47].

In contrast, patients’ WTU rebounded to 75.38% (1035/1373) in 2024, exceeding the levels recorded in both 2021 and 2022. This finding was also consistent with the logistic regression results from the pooled analysis, which showed a positive association between survey year 2024 and patients’ WTU. These findings suggested that although the pandemic no longer restricted patients’ choices of medical services, virtual visits may continue to serve as an important supplement to conventional health care in the postpandemic period. Several explanations may account for these findings. First, as the health care system returned to normal, most of the patients’ pent-up demand for in-person medical visits accumulated during the pandemic had been satisfied by 2024, allowing patients to re-evaluate the relative efficiency of different service modalities. By this time, the value of virtual visits had been widely recognized through patients’ pandemic-era experiences. Second, the habit of using virtual visits, formed during the pandemic, persisted among a subset of patients [48]. Consistent with this observation, Mandal et al [49] also found that, in the long term, after several years of the pandemic, the shift in health care delivery toward telemedicine was sustained and did not cease with the end of COVID-19. Third, recent advances in AI—including symptom recognition, intelligent triage, and auxiliary diagnosis [48]—have substantially improved the usability and usefulness of virtual visits. Virtual visit platforms have also gradually offered more diversified services and improved the patient experience [48]. Together, these factors may account for the high WTU observed in 2024.

Overall, these findings have important implications for the future development of virtual visits. As virtual visits transition from an emergency response to a routine component of health care delivery, future efforts should focus on integrating them into standard clinical practice rather than reserving them primarily for public health emergencies. Their application should be expanded to more appropriate clinical scenarios, and AI along with other digital technologies should be more deeply embedded into clinical workflows to enhance service quality and continuity of care. Supportive policies should continue to evolve, including refined reimbursement mechanisms, standardized service regulations, and strengthened data security and privacy protection. Collectively, these efforts may support the sustainable development of virtual visits in the postpandemic era.

#### Factors Associated With Patients’ WTU Virtual Visits During and After the COVID-19 Period

Both the pooled 2021-2024 analysis and the year-specific regression analyses identified similar factors associated with patients’ WTU, including age, education level, primary source of information, visiting hospital tier, and monthly income level. Previous studies have identified regional differences [50-52] and sex [27,50] as significant predictors of WTU telemedicine, whereas sex, marital status, and city income level were not associated with WTU in any survey year, in either the pooled or the year-specific models. The discrepancy regarding regional differences may be explained by differences in study settings. Zhejiang Province has relatively advanced digital health development and internet health care infrastructure in China, and our study focused exclusively on urban residents. These characteristics may have reduced differences in patients’ access to virtual visits across regions with different levels of economic development. In addition, the inconsistent findings regarding sex may be related to differences in sample composition.

Education level and primary source of information were the most stable correlates of WTU: they were the only variables significantly associated with WTU in 2021 and remained significant in every year-specific model and in the pooled-sample model. These findings were consistent with previous studies, reporting that patients with higher education levels were more likely to be willing to use virtual visits [50,53], that highly educated patients were generally more interested in using eHealth [54,55], and that patients whose primary source of information was the internet were more likely to be willing to use virtual visits [56]. One possible explanation was that patients’ educational level and reliance on the internet as the primary information source may, to some extent, reflect their digital literacy [57]. In addition, previous studies have suggested that digital literacy was an important prerequisite for the adoption of virtual visits [58].

Beyond these stable correlates, a wider range of variables was associated with WTU in the postpandemic waves. A possible interpretation is that, during the pandemic waves of 2021-2022, when in-person medical visits were restricted, virtual visits served as an important alternative channel of care, and WTU was primarily associated with education level and internet-based information seeking—both of which may reflect digital literacy. After the adjustment of China’s COVID-19 containment policies in 2023, in-person health services gradually resumed, virtual visits were no longer the only feasible option, and patients’ decisions may have increasingly reflected additional considerations such as technology accessibility, service quality, and convenience.

Consistent with this interpretation, older age was associated with lower WTU beginning in 2023, and the association extended to nearly all adult age groups in 2024; this aligned with previous studies identifying age as a significant factor associated with WTU telemedicine [50,52] and showing that younger and middle-aged patients were generally more receptive to emerging technologies and faced fewer barriers to using internet-based health services [50,59]. One possible contextual explanation was that, following the transition to the postpandemic period in 2023, patients had more health care options available than during the pandemic.

Visiting hospital tier was an additional factor significantly associated with WTU in 2022 and 2023. Patients attending nontertiary hospitals were less likely to be willing to use virtual visits than those attending tertiary hospitals. One possible explanation was that tertiary hospitals generally adopted internet hospitals earlier and offered more comprehensive online services, such as virtual visits, electronic prescriptions, and home medication delivery. They also tend to promote these services more actively. As a result, patients attending tertiary hospitals were more likely to be exposed to and gain experience with virtual visits, which may have increased their recognition of the convenience and effectiveness of this model of care.

In addition, medium-low monthly income, medium monthly income, and better self-rated health status were associated with higher WTU only in 2024, suggesting that payment capacity and health awareness became relevant to WTU in the postpandemic period. One possible explanation was that patients with better self-rated health status and higher income levels tended to pay greater attention to their own health and had a stronger demand for high-quality medical services [27,60], for which virtual visits offer a more convenient access channel to access high-quality care.

This study’s findings carry several implications. The sustained—and ultimately increasing—WTU virtual visits among outpatients suggests that demand for internet-hospital services can remain strong after the transition to routine pandemic management, supporting the continued integration of virtual visits into routine outpatient care in economically and digitally developed regions of China. Because education level and internet-based information seeking were the most stable correlates of WTU, health care institutions should further optimize virtual visit platforms with respect to usability and accessibility, with particular attention to patients with lower educational levels and limited internet skills; more convenient and user-friendly service design—such as simplified interfaces, multimodal guidance (eg, audio and video assistance), and on-site support at hospital outpatient departments—may help reduce digital barriers and increase WTU among these patients. In addition, the age association that emerged only in the postpandemic waves and progressively extended to younger patients suggested that acceptance barriers during the routine management era were not confined to older adults; outreach measures may therefore need to cover a broader range of age groups. Finally, the lower WTU observed among patients attending nontertiary hospitals in 2022-2023 suggests that these facilities may require additional support—such as clinician referral training and service promotion—if virtual visits are to reach patients across the full delivery system rather than primarily those attending tertiary hospitals.

### Limitations and Future Research

This study has several limitations. First, the sampling frame was not constant across waves: the 2021 survey covered Hangzhou only, whereas later waves extended across Zhejiang Province. In the Hangzhou-restricted sensitivity analysis, the elevated WTU in 2024 remained significant, whereas the 2023 estimate was attenuated and no longer significant. As Hangzhou’s digital development exceeds the provincial average, the 2021 baseline may have been relatively high, biasing subsequent estimates downward; the magnitude of the 2023 decline should therefore be interpreted with caution. Second, although the quota sampling design ensured proportionate representation by city income level, the distributions of other demographic characteristics may not have matched those of the target population, and recruiting only outpatients may have excluded individuals who did not seek in-person care during the survey periods. Future studies using community-based samples would improve representativeness. Third, the fieldwork periods varied across survey waves, and seasonal variation in outpatient characteristics and health care use may have reduced the comparability of findings across years. A sensitivity analysis restricted to the summer waves (2022 and 2024) yielded consistent findings, suggesting that seasonality is unlikely to explain the observed patterns; nevertheless, future studies should conduct surveys during comparable periods or across multiple seasons within each year.

Fourth, although the data had a hierarchical structure (patients nested within hospitals and hospitals within cities), the limited number of cities (G=9) prevented us from fully accounting for potential city-level clustering. Sensitivity analyses using hospital-clustered SEs and city fixed effects yielded generally consistent estimates. Future studies involving a larger number of cities should consider city-level clustering (eg, cluster-robust SEs). Fifth, patients’ WTU virtual visits was measured using a single-item question. Although intuitive and efficient, using validated multi-item scales in future research would provide a more robust and comprehensive assessment of patients’ behavioral intentions. Finally, the surveys were conducted among urban residents of Zhejiang, an economically and digitally developed province of China; given persistent urban-rural and regional disparities in economic development, health care access, and digital health adoption, the findings may not generalize to rural populations, less developed regions, or countries with different telemedicine delivery models.

### Conclusions

Patients’ WTU virtual visits remained high throughout 2021-2024, spanning China’s transition from stringent COVID-19 containment to routine pandemic management: WTU was stable during the pandemic years of 2021-2022, declined in the first postpandemic wave of 2023, and rebounded to its highest level in 2024. This temporal pattern suggests that demand for internet-hospital services can remain strong under routine pandemic management, supporting the continued integration of virtual visits into routine outpatient care. The correlates of WTU were only partially stable: a college-level education and internet-based information seeking were consistently associated with higher WTU in both the pooled and year-specific analyses, whereas the associations with age, visiting hospital tier, monthly income, and self-rated health status varied across survey years and emerged only in specific periods. These findings suggest that virtual visits gained sustained acceptance among patients in economically and digitally developed regions of China, while acceptance barriers in the postpandemic period extended beyond older adults. Future efforts should therefore prioritize improving the usability and accessibility of virtual visit platforms for patients with lower educational levels and limited internet skills, extending outreach to a broader range of age groups, providing additional support for nontertiary hospitals, and further integrating virtual visits into routine health care.

## Data Availability

The datasets generated and analyzed during this study are not publicly available due to participant privacy and ethical restrictions but are available from the corresponding author on reasonable request.

## Appendix

Multimedia Appendix 1 STROBE checklist.

Multimedia Appendix 2 Survey questionnaire.

Multimedia Appendix 3 Binary logistic regression results of patients’ willingness to use virtual visits based on the 2021-2024 repeated cross-sectional data: robust SE versus cluster‑robust SE.

Multimedia Appendix 4 Binary logistic regression results of patients’ willingness to use of virtual visits in 2023: robust SE vs cluster‑robust SE.

Multimedia Appendix 5 Binary logistic regression results of patients' willingness to use virtual visits in 2024: robust SE versus cluster‑robust SE.

Multimedia Appendix 6 Binary logistic regression results for patients’ willingness to use virtual visits based on the 2021-2024 repeated cross-sectional data with 9 city fixed effects and hospital-level cluster-robust SEs.

Multimedia Appendix 7 Sensitivity analysis restricted to the summer waves (2022 and 2024): pooled 2021-2024 versus summer-wave subsample logistic regression results.

## Abbreviations

GDP: gross domestic product
HINTS: Health Information National Trends Survey
OR: odds ratio
STROBE: Strengthening the Reporting of Observational Studies in Epidemiology
WTU: willingness to use
