# Supplementary material for: Chinese Patients’ Willingness to Use Virtual Visits During and After the COVID-19 Pandemic: Repeated Cross-Sectional Study in Zhejiang Province, 2021-2024
Source: J Med Internet Res. 2026 Oct 15;28:e87578. doi: 10.2196/87578 (PMC13720411; doi:10.2196/87578)
Supplement: Supplementary file 1 [file jmir_v28i1e87578_app1.doc]

STROBE Statement—Checklist of items that should be included in reports of ***cross-sectional studies***

|  | Item No | Recommendation | Section/Page No. |
| --- | --- | --- | --- |
| **Title and abstract** | 1 | (*a*) Indicate the study’s design with a commonly used term in the title or the abstract | Title and the Abstract (Page1-2) |
|  |  | (*b*) Provide in the abstract an informative and balanced summary of what was done and what was found | Title and Abstract (Page1-2) |
| Introduction | | |  |
| Background/rationale | 2 | Explain the scientific background and rationale for the investigation being reported | Introduction (Page 2-5) |
| Objectives | 3 | State specific objectives, including any prespecified hypotheses | Introduction (Page 5) |
| Methods | | |  |
| Study design | 4 | Present key elements of study design early in the paper | Method (Page 5-11) |
| Setting | 5 | Describe the setting, locations, and relevant dates, including periods of recruitment, exposure, follow-up, and data collection | Method (Page 5-11) |
| Participants | 6 | (*a*) Give the eligibility criteria, and the sources and methods of selection of participants | Method (Page 5-11) |
| Variables | 7 | Clearly define all outcomes, exposures, predictors, potential confounders, and effect modifiers. Give diagnostic criteria, if applicable | Method: Measurement  (Page 8-9) |
| Data sources/ measurement | 8* | For each variable of interest, give sources of data and details of methods of assessment (measurement). Describe comparability of assessment methods if there is more than one group | Method: Measurement  (Page 8-9) |
| Bias | 9 | Describe any efforts to address potential sources of bias | Method (Page 5, 8, 10); Result (Page 13-15; 18-22) |
| Study size | 10 | Explain how the study size was arrived at | Method: Participants and Data Collection  (Page 5-8); Limitation (Page 31-32) |
| Quantitative variables | 11 | Explain how quantitative variables were handled in the analyses. If applicable, describe which groupings were chosen and why | Method: Measurement (Page 8-9) |
| Statistical methods | 12 | (*a*) Describe all statistical methods, including those used to control for confounding | Method: Statistical Analysis Strategy (Page 10-11) |
|  |  | (*b*) Describe any methods used to examine subgroups and interactions | Method: Statistical Analysis Strategy (Page 10-11) |
|  |  | (*c*) Explain how missing data were addressed | Result: Figure 1: (Page 10) |
|  |  | (*d*) If applicable, describe analytical methods taking account of sampling strategy | Method: Statistical Analysis Strategy  (Page 10-11) |
|  |  | (*e*) Describe any sensitivity analyses | Method: Statistical Analysis Strategy  (Page 10-11) |
| Results | | |  |
| Participants | 13* | (a) Report numbers of individuals at each stage of study—eg numbers potentially eligible, examined for eligibility, confirmed eligible, included in the study, completing follow-up, and analysed | Method (Page 9-10) |
|  |  | (b) Give reasons for non-participation at each stage | Method (Page 9-10) |
|  |  | (c) Consider use of a flow diagram | Result: Figure 1: (Page 10) |
| Descriptive data | 14* | (a) Give characteristics of study participants (eg demographic, clinical, social) and information on exposures and potential confounders | Result: Table 2: (Page 12-13) |
|  |  | (b) Indicate number of participants with missing data for each variable of interest | Result: Figure 1: (Page 10) |
| Outcome data | 15* | Report numbers of outcome events or summary measures | Result: (Page 13-20) |
| Main results | 16 | (*a*) Give unadjusted estimates and, if applicable, confounder-adjusted estimates and their precision (eg, 95% confidence interval). Make clear which confounders were adjusted for and why they were included | Results (Page 10-23); Multimedia Appendix (Appendix 3) |
|  |  | (*b*) Report category boundaries when continuous variables were categorized | Results (Page 14-15; 17-18) |
|  |  | (*c*) If relevant, consider translating estimates of relative risk into absolute risk for a meaningful time period | N/A |
| Other analyses | 17 | Report other analyses done—eg analyses of subgroups and interactions, and sensitivity analyses | Result: Sensitivity Analysis  (Page 20-23) |
| Discussion | | |  |
| Key results | 18 | Summarise key results with reference to study objectives | Discussion:  Principal Findings  (Page 23-24) |
| Limitations | 19 | Discuss limitations of the study, taking into account sources of potential bias or imprecision. Discuss both direction and magnitude of any potential bias | Limitations and Future Research  (Page 29) |
| Interpretation | 20 | Give a cautious overall interpretation of results considering objectives, limitations, multiplicity of analyses, results from similar studies, and other relevant evidence | Discussion: Comparison With Prior Work  (Page 22-29) |
| Generalisability | 21 | Discuss the generalisability (external validity) of the study results | Discussion, Limitations and future research (Page 31-32) |
| Other information | | |  |
| Funding | 22 | Give the source of funding and the role of the funders for the present study and, if applicable, for the original study on which the present article is based | Funding (Page 32) |

*Give information separately for exposed and unexposed groups.
