# Supplementary material for: Chinese Patients’ Willingness to Use Virtual Visits During and After the COVID-19 Pandemic: Repeated Cross-Sectional Study in Zhejiang Province, 2021-2024
Source: J Med Internet Res. 2026 Oct 15;28:e87578. doi: 10.2196/87578 (PMC13720411; doi:10.2196/87578)
Supplement: Supplementary file 2 [file jmir_v28i1e87578_app2.doc]

English Version:

**Survey on Zhejiang Residents’ adoption behaviour of internet hospital services**

**Hello!** We are students from Zhejiang Chinese Medical University. We are conducting this survey to better understand Zhejiang residents’ adoption of **internet hospital services** and their preference. This questionnaire is anonymous, does not involve your personal privacy, and the results will be used for academic research only. Your participation is entirely voluntary, and you may withdraw at any time. By completing and returning this questionnaire, you are deemed to have consented to take part. Please feel free to complete it honestly. Please tick the option that best matches your actual situation. Thank you for your support and cooperation!

**1. What is your gender?**

A. Male	B. Female

**2. What is your age?**

A. Under 18 years old	B. 18–29 years old	C. 30–40 years old

D. 41–55 years old	E. 56–65 years old	F. Over 65 years old

**3. How would you rate your health status?**
A. Very poor	B. Poor	C. Fair	D. Good	E. Very good

**4. What is your marital status?**
A. Married	B. Single	C. Divorced	D. Widowed

**5. What is your highest level of education?**
A. ≤ Junior high school	B. Senior high school	C. College graduate	D. Postgraduate

**6. What is your approximate monthly income? (For reference, the conversion rate was CNY 1=US $0.149 as of September 21, 2026)**

A. Low income (<1,800 CNY/month in 2021–2023; <2,000 CNY/month in 2024)

B. Medium low income (1,801–5,000 CNY/month in 2021–2023; 2,001–5,000

CNY/month in 2024)

C. Medium income (5,001–8,000 CNY/month in 2021–2024)

D. Medium high income (8,001–17,000 CNY/month in 2021–2024)

E. High income (>17,000 CNY/month in 2021–2024)

**7. Is the internet your major source of information?**
A. No	B. Yes

**8. At present, telemedicine platforms can provide virtual visits, allowing physicians to diagnose and treat certain common and chronic conditions for non-first-visit patients, issue prescriptions, and deliver medications directly to patients’ homes. Do you know such services?**
A. No	B. Yes

**9. Would you be willing to use virtual visits?**
A. No	B. Yes

**10. Have you ever used virtual visits?**
A. No	B. Yes

Chinese Version:

**浙江省居民互联网医疗服务使用状况调查**

您好！我们是浙江中医药大学的学生。为了解浙江省居民对互联网医疗服务的采纳及相关需求，现开展一项关于浙江省居民互联网医疗服务使用的调查。本问卷采用匿名方式填写，不涉及您的个人隐私，调查结果仅用于学术研究。您的参与完全自愿，可随时退出。填写并交回本问卷即视为同意参与。请您根据实际情况，在符合的选项前打勾。感谢您的支持与配合！

1、您的性别是？

A、男	B、女

2、您的年龄是？

A、18岁以下	B、18-29岁	C、30-40岁	D、41-55岁	E、56-65岁	F、65岁以上

3、您觉得自己的健康状况怎么样？

A、很不好	B、不好	C、一般	D、好	E、很好

4、您的婚姻状况是？

A、已婚	B、未婚	C、离异	D、丧偶

5、您的受教育程度是？

A、初中及初中以下	B、高中	C、大学本科/专科	D、硕士研究生及以上

6、您的月收入大概是多少?（截至2026年9月21日，汇率为1人民币=0.149美元。）

A、低收入（2021—2023年：<1,800元/月；2024年：<2,000元/月）

B、中低收入（2021—2023年：1,801—5,000元/月；2024年：2,001—5,000元/月）

C、中等收入（2021—2024年：5,001—8,000元/月）

D、中高收入（2021—2024年：8,001—17,000元/月）

E、高收入（2021—2024年：>17,000元/月）

7、互联网是您主要的信息来源吗？

A、不是	B、是

8、现在互联网医院可以开展部分常见病、慢性病的在线复诊（网上复诊、开电子处方、送药上门），您知道吗？

A、不知道	B、知道

9、您愿意使用在线复诊吗？

A、不愿意	B、愿意

10、您有用过在线复诊吗？

A、没有	B、有
