# Supplementary material for: Chinese Patients’ Willingness to Use Virtual Visits During and After the COVID-19 Pandemic: Repeated Cross-Sectional Study in Zhejiang Province, 2021-2024
Source: J Med Internet Res. 2026 Oct 15;28:e87578. doi: 10.2196/87578 (PMC13720411; doi:10.2196/87578)
Supplement: Supplementary file 3 [file jmir_v28i1e87578_app3.doc]

**Table S1.** Binary logistic regression results of patients’ willingness to use virtual visits based on the 2021–2024 repeated cross-sectional data: robust SE vs. cluster‑robust SE

(N = 3678; McFadden pseudo-R² = 0.1365; Hosmer-Lemeshow χ²_8_ = 12.0, p = 0.15; Max VIF = 3.63)

| **Variable** | **Robust SE Model** | | **Clustered SE Model** | |
| --- | --- | --- | --- | --- |
|  | **OR (95%CI)** | **Standard error of coefficient** | **OR (95%CI)** | **Standard error of coefficient** |
| **Gender (ref: Male)** | | | | |
| Female | 1.15 (0.97–1.37) | 0.099 | 1.15 (0.98–1.35) | 0.095 |
| **Age group (ref: 18–29)** | | | | |
| 30–40 | 0.94 (0.71–1.24) | 0.133 | 0.94 (0.72–1.23) | 0.129 |
| 41–55 | 0.76 (0.56–1.02) | 0.116 | 0.76 (0.56–1.02) | 0.114 |
| 56–65 | 0.40 (0.28–0.57)*** | 0.072 | 0.40 (0.27–0.59)*** | 0.078 |
| > 65 | 0.26 (0.17–0.40)*** | 0.057 | 0.26 (0.15–0.44)*** | 0.069 |
| **Self-rated health status** | | | | |
|  | 1.04 (0.94–1.15) | 0.055 | 1.04 (0.92–1.18) | 0.066 |
| **Marital status (ref: Married)** | | | | |
| Single | 1.24 (0.94–1.65) | 0.178 | 1.24 (0.92–1.68) | 0.190 |
| Divorced | 0.82 (0.43–1.57) | 0.272 | 0.82 (0.37–1.80) | 0.329 |
| Widowed | 0.72 (0.19–2.78) | 0.497 | 0.72 (0.15–3.42) | 0.573 |
| **Education level (ref: ≤ Junior high school)** | | | | |
| Senior high school | 1.72 (1.36–2.18)*** | 0.207 | 1.72 (1.35–2.19)*** | 0.211 |
| College graduate | 2.74 (2.16–3.48)*** | 0.332 | 2.74 (2.21–3.40)*** | 0.302 |
| Postgraduate | 3.10 (1.93–4.98)*** | 0.749 | 3.10 (2.03–4.76)*** | 0.675 |
| **Monthly income level (CNY; ref: Low income)** | | | | |
| Medium low income | 1.27 (0.99–1.63) | 0.162 | 1.27 (1.07–1.51)** | 0.111 |
| Medium income | 1.36 (1.04–1.77)* | 0.185 | 1.36 (1.08–1.70)** | 0.156 |
| Medium high income | 1.08 (0.80–1.46) | 0.165 | 1.08 (0.83–1.42) | 0.150 |
| High income | 1.25 (0.84–1.87) | 0.256 | 1.25 (0.83–1.88) | 0.261 |
| **Primary source of information (ref: Non-Internet)** | | | | |
| Internet | 1.70 (1.42–2.03)*** | 0.155 | 1.70 (1.44–2.00)*** | 0.140 |
| **Hospital tier (ref: Tertiary hospitals)** | | | | |
| Non-tertiary hospitals | 0.80 (0.66–0.97)* | 0.079 | 0.80 (0.63–1.02) | 0.097 |
| **City income level (ref: High-income cities)** | | | | |
| Medium-income cities | 0.92 (0.73–1.14) | 0.103 | 0.92 (0.70–1.20) | 0.125 |
| Low-income cities | 0.97 (0.81–1.17) | 0.091 | 0.97 (0.75–1.26) | 0.128 |
| **Year (ref: 2021)** | | | | |
| 2022 | 1.19 (0.85–1.67) | 0.205 | 1.19 (0.80–1.78) | 0.245 |
| 2023 | 0.71 (0.52–0.97)* | 0.114 | 0.71 (0.51–0.99)* | 0.121 |
| 2024 | 1.42 (1.04–1.94)* | 0.227 | 1.42 (1.03–1.95)* | 0.230 |
| **Constant** | 0.71 (0.41–1.22) | 0.197 | 0.71 (0.45–1.12) | 0.164 |

*Note: Robust SE: Heteroskedasticity-robust standard errors. Clustered SE: Cluster-robust standard errors (clustered at hospital level). Clustered SE and robust SE yielded similar values, indicating weak within-hospital correlation. Robust SE is reported as the main specification. *: P < .050; **: P < .010; ***: P < .001*
