# Supplementary material for: Chinese Patients’ Willingness to Use Virtual Visits During and After the COVID-19 Pandemic: Repeated Cross-Sectional Study in Zhejiang Province, 2021-2024
Source: J Med Internet Res. 2026 Oct 15;28:e87578. doi: 10.2196/87578 (PMC13720411; doi:10.2196/87578)
Supplement: Supplementary file 4 [file jmir_v28i1e87578_app4.doc]

**Table S1.** Binary logistic regression results of patients’ willingness to use virtual visits in 2023: robust SE vs. cluster‑robust SE

(N = 1179; McFadden pseudo-R² = 0.1575; Hosmer-Lemeshow χ²_8_ = 2.6, p = 0.96; Max VIF = 3.03)

| **Variable** | **Robust SE** | | **Cluster SE** | |
| --- | --- | --- | --- | --- |
|  | **OR (95%CI)** | **Standard error of coefficient** | **OR (95%CI)** | **Standard error of coefficient** |
| **Gender (ref: Male)** | | | | |
| Female | 1.18 (0.88–1.59) | 0.179 | 1.18 (0.87–1.60) | 0.184 |
| **Age group (ref: 18–29)** | | | | |
| 30–40 | 0.92 (0.58–1.44) | 0.210 | 0.92 (0.59–1.43) | 0.207 |
| 41–55 | 0.67 (0.41–1.11) | 0.172 | 0.67 (0.44–1.04) | 0.149 |
| 56–65 | 0.35 (0.19–0.64)** | 0.108 | 0.35 (0.17–0.72)** | 0.130 |
| > 65 | 0.35 (0.17–0.72)** | 0.129 | 0.35 (0.17–0.74)** | 0.132 |
| **Self-rated health status** | | | | |
|  | 0.99 (0.83–1.19) | 0.090 | 0.99 (0.80–1.24) | 0.111 |
| **Marital status (ref: Married)** | | | | |
| Single | 1.37 (0.87–2.17) | 0.320 | 1.37 (0.82–2.30) | 0.360 |
| Divorced | 0.40 (0.10–1.59) | 0.280 | 0.40 (0.12–1.33) | 0.245 |
| Widowed | 3.42 (0.69–17.05) | 2.803 | 3.42 (0.76–15.39) | 2.625 |
| **Education level (ref: ≤ Junior high school)** | | | | |
| Senior high school | 1.84 (1.20–2.83)** | 0.404 | 1.84 (1.28–2.66)** | 0.345 |
| College graduate | 2.84 (1.86–4.36)*** | 0.620 | 2.84 (2.03–3.98)*** | 0.488 |
| Postgraduate | 4.26 (1.83–9.89)** | 1.831 | 4.26 (1.58–11.45)** | 2.150 |
| **Monthly income level (CNY; ref: Low income)** | | | | |
| Medium low income | 0.97 (0.65–1.46) | 0.200 | 0.97 (0.70–1.35) | 0.162 |
| Medium income | 1.15 (0.74–1.80) | 0.262 | 1.15 (0.76–1.75) | 0.247 |
| Medium high income | 1.01 (0.62–1.66) | 0.256 | 1.01 (0.63–1.62) | 0.242 |
| High income | 0.96 (0.51–1.83) | 0.314 | 0.96 (0.57–1.64) | 0.260 |
| **Primary source of information (ref: Non-Internet)** | | | | |
| Internet | 1.92 (1.39–2.65)*** | 0.317 | 1.92 (1.36–2.70)*** | 0.336 |
| **Hospital tier (ref: Tertiary hospitals)** | | | | |
| Non-tertiary hospitals | 0.65 (0.46–0.92)* | 0.114 | 0.65 (0.49–0.87)** | 0.095 |
| **City income level (ref: High-income cities)** | | | | |
| Medium-income cities | 0.90 (0.63–1.30) | 0.166 | 0.90 (0.62–1.31) | 0.171 |
| Low-income cities | 1.15 (0.85–1.57) | 0.180 | 1.15 (0.78–1.70) | 0.229 |
| **Constant** | 0.56 (0.24–1.30) | 0.241 | 0.56 (0.23–1.34) | 0.249 |

*Note: Robust SE: Heteroskedasticity-robust standard errors. Clustered SE: Cluster-robust standard errors (clustered at hospital level). Clustered SE and robust SE yielded similar values, indicating weak within-hospital correlation. Robust SE is reported as the primary specification. *: P < .050; **: P < .010; ***: P < .001*
