# Supplementary material for: Chinese Patients’ Willingness to Use Virtual Visits During and After the COVID-19 Pandemic: Repeated Cross-Sectional Study in Zhejiang Province, 2021-2024
Source: J Med Internet Res. 2026 Oct 15;28:e87578. doi: 10.2196/87578 (PMC13720411; doi:10.2196/87578)
Supplement: Supplementary file 5 [file jmir_v28i1e87578_app5.doc]

**Table S1.** Binary logistic regression results of patients’ willingness to use virtual visits in 2024: robust SE vs. cluster‑robust SE

(N = 1373; McFadden pseudo-R² = 0.1990; Hosmer-Lemeshow χ²_8_ = 12.1, p = 0.15; Max VIF = 3.27)

| **Variable** | **Robust SE** | | **Cluster SE** | |
| --- | --- | --- | --- | --- |
|  | **OR (95%CI)** | **Standard error of coefficient** | **OR (95%CI)** | **Standard error of coefficient** |
| **Gender (ref: Male)** | | | | |
| Female | 1.26 (0.92–1.72) | 0.201 | 1.26 (0.88–1.80) | 0.230 |
| **Age group (ref: 18–29)** | | | | |
| 30–40 | 0.59 (0.35–0.99)* | 0.156 | 0.59 (0.37–0.94)* | 0.139 |
| 41–55 | 0.51 (0.29–0.89)* | 0.145 | 0.51 (0.28–0.93)* | 0.157 |
| 56–65 | 0.25 (0.13–0.49)*** | 0.085 | 0.25 (0.15–0.44)*** | 0.072 |
| > 65 | 0.05 (0.02–0.12)*** | 0.023 | 0.05 (0.02–0.13)*** | 0.023 |
| **Self-rated health status** | | | | |
|  | 1.37 (1.13–1.67)** | 0.139 | 1.37 (1.15–1.63)*** | 0.122 |
| **Marital status (ref: Married)** | | | | |
| Single | 1.08 (0.64–1.85) | 0.295 | 1.08 (0.67–1.75) | 0.266 |
| Divorced | 1.46 (0.56–3.79) | 0.710 | 1.46 (0.45–4.71) | 0.873 |
| Widowed | 0.19 (0.01–5.89) | 0.335 | 0.19 (0.01–6.27) | 0.342 |
| **Education level (ref: ≤ Junior high school)** | | | | |
| Senior high school | 2.09 (1.37–3.20)** | 0.453 | 2.09 (1.39–3.15)*** | 0.436 |
| College graduate | 3.41 (2.28–5.10)*** | 0.701 | 3.41 (2.22–5.25)*** | 0.749 |
| Postgraduate | 3.21 (1.46–7.08)** | 1.296 | 3.21 (1.64–6.30)** | 1.105 |
| **Monthly income level (CNY; ref: Low income)** | | | | |
| Medium low income | 1.68 (1.04–2.70)* | 0.407 | 1.68 (1.19–2.35)** | 0.291 |
| Medium income | 1.74 (1.08–2.80)* | 0.423 | 1.74 (1.02–2.95)* | 0.468 |
| Medium high income | 1.27 (0.73–2.22) | 0.361 | 1.27 (0.73–2.22) | 0.361 |
| High income | 2.00 (0.95–4.19) | 0.755 | 2.00 (0.92–4.34) | 0.791 |
| **Primary source of information (ref: Non-Internet)** | | | | |
| Internet | 1.45 (1.07–1.97)* | 0.228 | 1.45 (1.08–1.94)* | 0.216 |
| **Hospital tier (ref: Tertiary hospitals)** | | | | |
| Non-tertiary hospitals | 1.20 (0.81–1.78) | 0.242 | 1.20 (0.71–2.01) | 0.317 |
| **City income level (ref: High-income cities)** | | | | |
| Medium-income cities | 0.84 (0.57–1.23) | 0.165 | 0.84 (0.57–1.23) | 0.165 |
| Low-income cities | 0.79 (0.57–1.10) | 0.132 | 0.79 (0.60–1.04) | 0.111 |
| **Constant** | 0.57 (0.22–1.49) | 0.278 | 0.57 (0.24–1.38) | 0.256 |

*Note: Robust SE: Heteroskedasticity-robust standard errors. Clustered SE: Cluster-robust standard errors (clustered at hospital level). Clustered SE and robust SE yielded similar values, indicating weak within-hospital correlation. Robust SE is reported as the primary specification. *: P < .050; **: P < .010; ***: P < .001*
