# Supplementary material for: Chinese Patients’ Willingness to Use Virtual Visits During and After the COVID-19 Pandemic: Repeated Cross-Sectional Study in Zhejiang Province, 2021-2024
Source: J Med Internet Res. 2026 Oct 15;28:e87578. doi: 10.2196/87578 (PMC13720411; doi:10.2196/87578)
Supplement: Supplementary file 6 [file jmir_v28i1e87578_app6.doc]

**Table S1.** Binary logistic regression results for patients’ willingness to use virtual visits based on the 2021–2024 repeated cross-sectional data with nine city fixed effects and hospital-level cluster-robust standard errors

(N = 3678; McFadden pseudo-R² = 0.1379; Hosmer-Lemeshow χ²_8_ = 5.6,, p = 0.69; Max VIF = 4.01)

| **Variable** | **OR (95%CI)** |
| --- | --- |
| **Gender (ref: Male)** | |
| Female | 1.15 (0.99–1.35) |
| **Age (ref: 18–29)** | |
| 30–40 | 0.95 (0.73–1.25) |
| 41–55 | 0.77 (0.57–1.03) |
| 56–65 | 0.40 (0.27–0.58)*** |
| > 65 | 0.26 (0.15–0.43)*** |
| **Self-rated health status** | |
|  | 1.04 (0.92–1.18) |
| **Marital status (ref: Married)** | |
| Single | 1.24 (0.92–1.68) |
| Divorced | 0.84 (0.39–1.84) |
| Widowed | 0.72 (0.15–3.40) |
| **Education level (ref: ≤ Junior high school)** | |
| Senior high school | 1.72 (1.35–2.18)*** |
| College graduate | 2.71 (2.18–3.36)*** |
| Postgraduate | 3.05 (2.01–4.64)*** |
| **Monthly income (CNY; ref: Low income)** | |
| Medium low income | 1.26 (1.05–1.52)* |
| Medium income | 1.34 (1.07–1.69)* |
| Medium high income | 1.07 (0.82–1.41) |
| High income | 1.21 (0.80–1.85) |
| **Primary source of information (ref: Non-Internet)** | |
| Internet | 1.70 (1.45–1.99)*** |
| **Hospital tier (ref: Tertiary hospitals)** | |
| Non-tertiary hospitals | 0.76 (0.61–0.96)* |
| **Year (ref: 2021)** | |
| 2022 | 1.23 (0.82–1.85) |
| 2023 | 0.75 (0.54–1.04) |
| 2024 | 1.50 (1.06–2.13)* |
| **City fixed effects** | Yes |
| Constant | 0.72 (0.45–1.14) |

**: P < .050; **: P < .010; ***: P < .001*
