# Supplementary material for: Chinese Patients’ Willingness to Use Virtual Visits During and After the COVID-19 Pandemic: Repeated Cross-Sectional Study in Zhejiang Province, 2021-2024
Source: J Med Internet Res. 2026 Oct 15;28:e87578. doi: 10.2196/87578 (PMC13720411; doi:10.2196/87578)
Supplement: Supplementary file 7 [file jmir_v28i1e87578_app7.doc]

**Table S1.** Sensitivity analysis restricted to the summer waves (2022 and 2024): pooled 2021–2024 versus summer-wave subsample logistic regression results

(2021–2024: N = 3678; McFadden pseudo-R² = 0.1365; Hosmer-Lemeshow χ²_8_ = 12.0, p = 0.15; Max VIF = 3.63; 2022 and 2024: N = 2159; McFadden pseudo-R² = 0.1314; Hosmer-Lemeshow χ²_8_ = 7.9, p = 0.44; Max VIF = 2.90)

| **Variable** | **2021**–**2024** | **2022 and 2024** |
| --- | --- | --- |
|  | **OR (95%CI)** | **OR (95%CI)** |
| **Gender (ref: Male)** | | |
| Female | 1.15 (0.97–1.37) | 1.15 (0.92–1.45) |
| **Age group (ref: 18–29)** | | |
| 30–40 | 0.94 (0.71–1.24) | 0.88 (0.60–1.29) |
| 41–55 | 0.76 (0.56–1.02) | 0.67 (0.44–1.01) |
| 56–65 | 0.40 (0.28–0.57)*** | 0.41 (0.25–0.67)*** |
| > 65 | 0.26 (0.17–0.40)*** | 0.16 (0.09–0.30)*** |
| **Self-rated health status** | | |
|  | 1.04 (0.94–1.15) | 1.14 (0.99–1.32) |
| **Marital status (ref: Married)** | | |
| Single | 1.24 (0.94–1.65) | 1.12 (0.76–1.65) |
| Divorced | 0.82 (0.43–1.57) | 0.88 (0.41–1.90) |
| Widowed | 0.72 (0.19–2.78) | 0.30 (0.04–2.06) |
| **Education level (ref: ≤ Junior high school)** | | |
| Senior high school | 1.72 (1.36–2.18)*** | 1.58 (1.16–2.15)** |
| College graduate | 2.74 (2.16–3.48)*** | 2.68 (1.96–3.66)*** |
| Postgraduate | 3.10 (1.93–4.98)*** | 2.74 (1.44–5.20)** |
| **Monthly income level(CNY; ref: Low income)** | | |
| Medium low income | 1.27 (0.99–1.63) | 1.54 (1.07–2.19)* |
| Medium income | 1.36 (1.04–1.77)* | 1.53 (1.05–2.22)* |
| Medium high income | 1.08 (0.80–1.46) | 1.24 (0.82–1.90) |
| High income | 1.25 (0.84–1.87) | 1.74 (0.96–3.14) |
| **Primary source of information (ref: Non-Internet)** | | |
| Internet | 1.70 (1.42–2.03)*** | 1.50 (1.19–1.90)** |
| **Hospital tier (ref: Tertiary hospitals)** | | |
| Non-tertiary hospitals | 0.80 (0.66–0.97)* | 0.82 (0.63–1.07) |
| **City income level (ref: High-income cities)** | | |
| Medium-income cities | 0.92 (0.73–1.14) | 0.88 (0.65–1.19) |
| Low-income cities | 0.97 (0.81–1.17) | 0.85 (0.67–1.08) |
| **Year (ref: 2021)** | | **Year (ref: 2022)** |
| 2022 | 1.19 (0.85–1.67) | / |
| 2023 | 0.71 (0.52–0.97)* | / |
| 2024 | 1.42 (1.04–1.94)* | 1.19 (0.94–1.50) |
| **Constant** | 0.71 (0.41–1.12) | 0.75 (0.36–1.57) |

*The results are based on heteroskedasticity-robust standard errors*. **: P < .050; **: P < .010; ***: P < .001*
